# Analysis of Rituximab Use, Time Between Rituximab and SARS-CoV-2 Vaccination, and COVID-19 Hospitalization or Death in Patients With Multiple Sclerosis

**DOI:** 10.1001/jamanetworkopen.2022.48664

**Published:** 2022-12-28

**Authors:** Jessica B. Smith, Edlin G. Gonzales, Bonnie H. Li, Annette Langer-Gould

**Affiliations:** 1 Department of Research & Evaluation, Southern California Permanente Medical Group, Pasadena; 2 Department of Neurology, Los Angeles Medical Center, Southern California Permanente Medical Group, Los Angeles

## Abstract

**Importance:**

Rituximab and other B-cell–depleting therapies blunt humoral responses to SARS-CoV-2 vaccines, particularly when the vaccine is administered within 6 months of an infusion. Whether this translates into an increased risk of hospitalization or death from COVID-19 is unclear.

**Objectives:**

To examine whether rituximab treatment is associated with an increased risk of hospitalization for COVID-19 among SARS-CoV-2–vaccinated persons with multiple sclerosis (MS) and whether delaying vaccination more than 6 months after rituximab treatment is associated with decreased risk.

**Design, Setting, and Participants:**

This retrospective cohort study used Kaiser Permanente Southern California’s electronic health record to identify individuals from January 1, 2020, to February 15, 2022, who had MS and who had been vaccinated against SARS-CoV-2.

**Exposures:**

Rituximab treatment compared with disease-modifying therapies (DMTs) that do not interfere with vaccine efficacy or being untreated (no or other DMT group). Among rituximab-treated patients, the exposure was receiving at least 1 vaccine dose more than 6 months after their last infusion compared with receiving all vaccine doses 6 months or less since their last infusion.

**Main Outcomes and Measures:**

The main outcome was hospitalization due to COVID-19 infection. The odds of infection resulting in hospitalization following SARS-CoV-2 vaccination were adjusted for race and ethnicity, advanced MS-related disability; vaccine type; booster dose; and, among rituximab-treated only analyses, cumulative rituximab dose and dose at last infusion. Exposures, outcomes, and covariates were collected from the electronic health record.

**Results:**

Among 3974 SARS-CoV-2–vaccinated people with MS (mean [SD] age, 55.3 [15] years; 2982 [75.0%] female; 103 [2.6%] Asian or Pacific Islander; 634 [16.0%] Black; 953 [24.0%] Hispanic; 2269 [57.1%] White; and 15 [0.3%] other race or ethnicity), rituximab-treated patients (n = 1516) were more likely to be hospitalized (n = 27) but not die (n = 0) compared with the 2458 individuals with MS receiving no or other DMTs (n = 7 and n = 0, respectively; adjusted odds ratio [aOR] for hospitalization, 7.33; 95% CI, 3.05-17.63). Receiving messenger RNA (mRNA) SARS-CoV-2 vaccine (aOR, 0.36; 95% CI, 0.15-0.90; *P* = .03) and receiving a booster vaccination (aOR, 0.31; 95% CI, 0.15-0.64; *P* = .002) were independently associated with a decreased risk of hospitalization for COVID-19. Among vaccinated rituximab-treated individuals with MS, receiving any vaccination dose more than 6 months after the last rituximab infusion was associated with a reduced risk of COVID-19 hospitalization (aOR, 0.22; 95% CI, 0.10-0.49).

**Conclusions and Relevance:**

This cohort study’s findings suggest that rituximab-treated people with MS should be strongly encouraged to receive mRNA SARS-CoV-2 vaccines and boosters more than 6 months after their last rituximab infusion whenever possible. The low absolute risk of hospitalization for COVID-19 among mRNA-vaccinated individuals with MS should not preclude use of rituximab, which has marked efficacy, cost, and convenience advantages over other DMTs.

## Key Points

**Question:**

Is rituximab treatment associated with an increased risk of hospitalization or death from COVID-19 among SARS-CoV-2–vaccinated persons with multiple sclerosis (MS), and is vaccinating patients more than 6 months after their last infusion associated with decreased risk?

**Findings:**

In this cohort study of 3974 SARS-CoV-2–vaccinated individuals with MS, the risk of hospitalization due to COVID-19 in those treated with rituximab was low (1.81%) but significantly higher compared with those who received no or other treatments (0.33%). Vaccinating rituximab-treated individuals with MS more than 6 months after their last infusion and receiving messenger RNA vaccines and boosters was associated with significant reduction in this risk.

**Meaning:**

These findings suggest that persons with MS who are receiving rituximab treatment should be strongly encouraged to receive messenger RNA SARS-CoV-2 vaccines and boosters, ideally more than 6 months after their last infusion.

## Teaser

This cohort study of SARS-CoV-2–vaccinated persons with multiple sclerosis examines the association between rituximab treatment and the risk of hospitalization for COVID-19.

## Introduction

Treatment with rituximab or other B-cell–depleting therapies has been associated with an increased risk of more severe COVID-19, including death in unvaccinated persons with multiple sclerosis (MS) compared with those treated with several other disease modifying-therapies (DMTs) and the general population without MS.^1,2,3^ A recent meta-analysis^4^ of SARS-CoV-2 vaccine responses among DMT-treated individuals with MS found that humoral but not T-cell–mediated immune responses were blunted among patients treated with B-cell–depleting therapies. This blunted humoral immune response was most pronounced in patients who were vaccinated within 6 months compared with those who were vaccinated more than 6 months after their last B-cell–depleting therapy infusion. Whether such blunted humoral vaccine immune responses translate into an increased risk of moderate or severe COVID-19 following vaccination, particularly if vaccines were administered more than 6 months after the last infusion in individuals with MS treated with rituximab or other B-cell–depleting therapies, is unclear.

The few studies^5,6,7^ to examine the risk of any breakthrough COVID-19 infections following vaccination in persons with MS receiving B-cell–depleting therapies have reported an increased risk compared with those receiving most other DMTs. These studies^5,6,7^ were unable to determine whether extending the dosing intervals of B-cell–depleting therapies could diminish this risk or whether this risk extends to more severe COVID-19, because few patients required hospitalization. The goal of this study was to determine whether rituximab treatment is associated with an increased risk of hospitalization for COVID-19 among SARS-CoV-2–vaccinated persons with MS and, if so, whether delaying vaccination more than 6 months after the most recent rituximab infusion is associated with decreased risk.

## Methods

We conducted a retrospective cohort study using Kaiser Permanente Southern California’s (KPSC’s) complete electronic health records (EHRs). The KPSC system provides care to more than 4.8 million members in Southern California, representing approximately 20% of the general population in the geographic areas served. The sociodemographic characteristics of the KPSC members are generally representative of the underlying population.^8^ The study protocol was approved by the KPSC Institutional Review Board with a waiver of informed consent because of the no more than minimal risk to the participants and the use of data that have already been collected in the EHRs. The study followed the Strengthening the Reporting of Observational Studies in Epidemiology (STROBE) reporting guideline.

The EHRs were electronically searched from January 1, 2020, to February 15, 2022, to identify the following: individuals with MS, start and stop dates of DMTs, clinical and demographic characteristics, SARS-CoV-2 infection, and COVID-19 course. The EHRs of rituximab-treated persons were reviewed to confirm MS diagnosis.^9,10^ Inclusion criteria for the main analyses were (1) diagnosis of MS using a validated algorithm^11^ and (2) SARS-CoV-2 vaccination. Individuals with MS were considered vaccinated 14 days after the second dose of messenger RNA (mRNA) vaccine (manufactured by Pfizer-BioNTech or Moderna) or first dose of viral vector vaccine (manufactured by Janssen/Johnson & Johnson) and boosted more than 14 days after a subsequent vaccine dose.^12^ Exclusion criteria were rituximab treatment for non-MS diagnoses (n = 219), treatment with DMTs known or suspected to interfere with vaccine efficacy during the study period (n = 80), or partial vaccination at index date (n = 65). SARS-CoV-2 infection required confirmation by polymerase chain reaction (PCR), positive antigen test results, or, to account for the widespread use of home antigen tests starting in December 2021, *International Statistical Classification of Diseases and Related Health Problems, Tenth Revision* (*ICD-10*) diagnostic code for acute COVID-19 infection.

The primary outcome was hospitalization for COVID-19 (eMethods in Supplement 1). The EHRs of hospitalized patients with MS with positive SARS-CoV-2 PCR test results were abstracted to determine whether the hospitalizations were due to COVID-19 or unrelated reasons (eg, labor and delivery) and to assess maximum severity of respiratory impairment.^13^ To capture all SARS-CoV-2 infections, we also included patients with mild COVID-19, defined as documented infection without requiring hospitalization. Characteristics were tabulated, and crude incidence rates of hospitalization for COVID-19 were calculated starting when the first individuals with MS received a COVID-19 vaccine (December 15, 2020).

Unconditional logistic regression was used to determine whether, among SARS-CoV-2–vaccinated persons with MS, rituximab use was associated with an increased odds of hospitalization for COVID-19 compared with persons with MS who were untreated during the study period or treated with DMTs that do not interfere with vaccine efficacy (natalizumab, glatiramer acetate, β-interferons, or dimethyl fumarate^4^), referred to hereafter as the no or other DMT group. The index date was defined as SARS-CoV-2 infection (n = 498) or, in the 3476 noninfected individuals, the end of the study period (February 15, 2022; n = 3303), membership termination date (n = 167), or date of death (n = 6), whichever came first. We examined factors previously identified as risk factors for moderate to severe COVID-19 in general or in MS populations^1^: age (continuous in years), sex (female or male), Elixhauser Comorbidity Index (continuous), advanced disability (walker or worse), vaccine type (mRNA or viral vector), booster dose (defined as receiving at least 1 additional vaccine dose following completion of the initial vaccination series), and time since last vaccine (≤150 or >150 days).^14^ Self-identified race and ethnicity categories, obtained from the EHRs, included American Indian or Alaska Native, Asian or Pacific Islander, Black, Hispanic, White, multiple races, and unknown. Outcomes by race and ethnicity were assessed as a surrogate measure for structural racism. Factors with *P* < .20 (to capture potential suppressor confounders) were included in the final adjusted model.

We used a similar approach to determine whether delaying vaccine administration more than 6 months after rituximab infusion was independently associated with COVID-19 hospitalizations among vaccinated persons with MS taking rituximab. We examined additional rituximab use characteristics previously implicated in COVID-19 severity, including dose at last infusion (≥1000 or <1000 mg) and cumulative dose (cut-off chosen at 95th percentile of ≥9000 mg). To assess whether people with MS taking rituximab may have been hospitalized despite having mild symptoms, sensitivity analyses excluding patients who did not require oxygen support during hospitalization were conducted. Because many individuals with MS in our population declined COVID-19 vaccinations, we extended our previous study^1^ to examine the risk of moderate to severe COVID-19 in unvaccinated vs vaccinated individuals with MS taking rituximab as a secondary analysis.

### Statistical Analysis

The means (SDs) of normally distributed variables were compared using 2-sample *t* tests. For variables with nonnormal distributions, the Wilcoxon rank-sum test was used. For binary or categorical variables, the χ^2^ test with the Fisher exact test was used. Statistical significance was set at a 2-sided *P* < .05. All statistical analyses were performed using SAS software, version 9.4 (SAS Institute Inc).

## Results

A total of 3974 SARS-CoV-2–vaccinated people with MS were analyzed (mean [SD] age, 55.3 [15] years; 2982 [75.0%] female and 992 [25.0%] male; 103 [2.6%] Asian or Pacific Islander; 634 [16.0%] Black; 953 [24.0%] Hispanic; 2269 [57.1%] White; and 15 [0.3%] other race or ethnicity [including American Indian or Alaska Native, other, or unknown]). The baseline demographic and clinical characteristics and COVID-19 outcomes in the rituximab group (n = 1516) compared with the no or other DMT group (n = 2458) are shown in Table 1. Individuals receiving rituximab treatment were slightly younger, were less likely to be White, had a lower Elixhauser Comorbidity Index, had slightly less disability, and were less likely to have received any booster vaccine doses during the study period compared with the no or other DMT group (Table 1). Most individuals with MS received mRNA vaccines (n = 3717 [93.5%]) in both groups. In the no or other DMT group, 232 were treated with interferon β, 216 with glatiramer acetate, 80 with natalizumab, and 30 with dimethyl fumarate, and 1900 were untreated.

**Table 1.** Patient Characteristics and COVID-19 Outcomes Among SARS-CoV-2–Vaccinated People With Multiple Sclerosis Stratified by Treatment Group^a^

Characteristic or outcome	Rituximab (n = 1516)	No or other DMT (n = 2458)^b^	Total (N = 3974)	*P* value
Demographic and clinical characteristics^c^				
Age, mean (SD), y	47.0 (12.4)	60.4 (14.2)	55.3 (15.0)	<.001
Sex				
Female	1116 (73.6)	1866 (75.9)	2982 (75.0)	.10
Male	400 (26.4)	592 (24.1)	992 (25.0)	
Race and ethnicity				
Asian or Pacific Islander	48 (3.2)	55 (2.2)	103 (2.6)	<.001
Black	254 (16.7)	380 (15.5)	634 (16.0)	
Hispanic	461 (30.4)	492 (20.0)	953 (24.0)	
White	741 (48.9)	1528 (62.2)	2269 (57.1)	
Other^d^	12 (0.8)	3 (0.1)	15 (0.3)	
Advanced disability (walker or worse)	245 (16.2)	458 (18.6)	703 (17.7)	.05
Elixhauser Comorbidity Index score, median (IQR)	2.0 (1.0-3.0)	2.0 (1.0-4.0)	2.0 (1.0-4.0)	<.001
Study follow-up time, mean (SD), y	2.1 (0.1)	2.1 (0.2)	2.1 (0.2)	.04
COVID-19 vaccination characteristics				
Vaccinated, not boosted	580 (38.3)	797 (32.4)	1377 (34.7)	<.001
Vaccinated and ≥1 booster or additional dose	936 (61.7)	1661 (67.6)	2597 (65.3)	
No. of additional vaccine doses				
0	580 (38.3)	797 (32.4)	1377 (34.7)	<.001
1	921 (60.7)	1653 (67.3)	2574 (64.7)	
2	12 (0.8)	8 (0.3)	20 (0.5)	
3	3 (0.2)	0	3 (0.1)	
Time since last vaccine dose, d				
>150	542 (35.8)	707 (28.8)	1249 (31.4)	<.001
≤150	974 (64.2)	1751 (71.2)	2725 (68.6)	
Time since last vaccine, dose, median (IQR), mo	3.8 (2.3-5.9)	3.6 (2.3-5.6)	3.7 (2.3-5.8)	.31
Vaccination types				
mRNA vaccines only	1417 (93.5)	2300 (93.6)	3717 (93.5)	.93
Viral vector vaccines only	72 (4.7)	118 (4.8)	190 (4.8)	
Viral vector followed by mRNA booster	27 (1.8)	40 (1.6)	67 (1.7)	
COVID-19 outcomes				
No infection	1232 (81.2)	2244 (91.3)	3476 (87.5)	<.001
COVID-19 but not hospitalized	257 (17.0)	207 (8.4)	464 (11.7)	
Hospitalized for COVID-19	27 (1.8)	7 (0.3)	34 (0.8)	
Severity of respiratory impairment among hospitalized patients				
No oxygen required	9 (0.6)	1 (0.04)	10 (0.3)	.40
Mild (>0 and <6 L/min via nasal cannula)	13 (0.9)	4 (0.2)	17 (0.4)	
Moderate (6-15 L/min via nasal cannula or 40%-60% via face mask)	0	1 (0.04)	1 (0.03)	
Severe (high-flow NIV >15 L/min via nasal cannula or >60% via face mask)	4 (0.3)	1 (0.04)	5 (0.1)	
Invasive mechanical ventilation	1 (0.07)	0	1 (0.03)	
Death	0	0	0	
Antiviral use				
None	1489 (98.2)	2448 (99.6)	3937 (99.1)	<.001
Nirmatrelvir-ritonavir	1 (0.1)	2 (0.1)	3 (0.1)	>.99
Remdesivir	26 (1.7)	8 (0.3)	34 (0.9)	<.001

Abbreviations: DMT, disease-modifying therapy; mRNA, messenger ribonucleic acid; NIV, noninvasive ventilation.

^a^ Data are presented as number (percentage) of study participants unless otherwise indicated.

^b^ Untreated persons with multiple sclerosis or treated with DMTs that do not interfere with SARS-CoV-2 vaccine efficacy (interferon betas, glatiramer acetate, natalizumab, or dimethyl fumarate).

^c^ At index date.

^d^ Other race and ethnicity includes American Indian or Alaska Native (n = 3), multiple races (n = 11), and unknown race (n = 1).

The proportion of persons with MS hospitalized for breakthrough COVID-19 infections was low in both groups but higher among those receiving rituximab treatment compared with the no or other DMT group (Table 1). Rituximab-treated individuals with COVID-19 were more likely to be hospitalized (n = 27 [1.8%]) compared with the no or other DMT group (n = 7 [0.3%]). Use of antivirals was rare and occurred most often in hospitalized individuals in the rituximab group (n = 16). Hospitalizations occurred more frequently during the Omicron variant outbreak (December 1, 2021, to February 15, 2022) than during the Delta variant outbreak (June 1, 2021, to November 30, 2021) among those receiving rituximab (n = 18 and n = 9, respectively) but were similar in the no or other DMT group (n = 3 and n = 4, respectively) (eTable 1 in Supplement 1). None of the vaccinated individuals with MS died of COVID-19. One rituximab-treated person with MS required mechanical ventilation and 4 others had severe respiratory impairment that required high-flow oxygen via face mask or noninvasive ventilation, compared with only 1 patient with severe respiratory impairment and none requiring mechanical ventilation in the no or other DMT group (Table 1). Of the 34 people with MS hospitalized for COVID-19, 6 (17.6%) had received a viral vector vaccine, all of whom were treated with rituximab.

In a mutually adjusted model, treatment with rituximab and greater Elixhauser Comorbidity Index scores were independently associated with an increased risk of hospitalization for COVID-19, and receiving a booster dose or mRNA vaccine was associated with a decreased risk (Table 2). Age, sex, and time since last vaccination were not associated with hospitalization for COVID-19, and ethnicity was no longer significant in adjusted models (Table 2).

**Table 2.** Association of Rituximab Treatment With COVID-19 Hospitalization Among SARS-CoV-2–Vaccinated Patients With MS

Variable	Rituximab compared with no or other DMT group^a^				Comparison of rituximab treatment characteristics among patients with MS treated with rituximab			
	Crude		Adjusted		Crude		Adjusted	
	OR (95% CI)	*P* value	OR (95% CI)	*P* value	OR (95% CI)	*P* value	OR (95% CI)	*P* value
Age,	0.99 (0.97-1.01)	.43	NA	NA	1.01 (0.98-1.04)	.58	NA	NA
Female sex	0.92 (0.43-1.99)	.84	NA	NA	0.85 (0.37-1.95)	.70	NA	NA
White race	0.46 (0.23-0.93)	.03	0.62 (0.31-1.26)	.18	0.52 (0.23-1.16)	.11	0.58 (0.25-1.35)	.21
Advanced disability (walker or worse)	1.21 (0.52-2.79)	.66	NA	NA	1.84 (0.77-4.40)	.17	1.82 (0.69-4.78)	.22
Elixhauser Comorbidity Index	1.10 (0.96-1.27)	.17	1.25 (1.08-1.45)	.003	1.10 (0.89-1.35)	.38	NA	NA
Vaccination characteristics								
mRNA vaccines only	0.32 (0.13-0.77)	.01	0.36 (0.15-0.90)	.03	0.22 (0.09-0.59)	.002	0.28 (0.11-0.73)	.009
≥1 Booster dose received	0.29 (0.14-0.58)	<.001	0.31 (0.15-0.64)	.002	0.49 (0.23-1.05)	.07	0.61 (0.28-1.34)	.22
≤150 Days since last vaccine	0.65 (0.33-1.30)	.22	NA	NA	1.60 (0.67-3.81)	.29	NA	NA
Rituximab treatment	6.35 (2.76-14.62)	<.001	7.33 (3.05-17.63)	<.001	NA	NA	NA	NA
Rituximab characteristics								
Timing of SARS-CoV-2 vaccine dose								
All doses ≤6 mo	NA	NA	NA	NA	1 [Reference]	NA	1 [Reference]	NA
Any dose >6 mo	NA	NA	NA	NA	0.17 (0.08-0.37)	<.001	0.22 (0.10-0.49)	<.001
Dose at last infusion ≥1000 mg	NA	NA	NA	NA	2.04 (0.69-6.00)	.20	1.11 (0.32-3.86)	.87
Cumulative dose ≥9000 mg	NA	NA	NA	NA	3.90 (1.44-10.56)	.007	2.50 (0.77-8.09)	.13

Abbreviations: DMT, disease-modifying therapy; mRNA, messenger RNA; MS, multiple sclerosis; NA, not applicable; OR, odds ratio.

^a^ Untreated persons with MS or treated with DMTs that do not interfere with SARS-CoV-2 vaccine efficacy (interferon-betas, glatiramer acetate, natalizumab, or dimethyl fumarate).

The demographic and clinical characteristics of vaccinated individuals with MS taking rituximab stratified by COVID-19 outcomes are presented in Table 3. Individuals who were hospitalized for COVID-19 were older, were less likely to be White, had more disability, and were less likely to have received a booster or any vaccine dose more than 6 months after their last rituximab infusion compared with those without any COVID-19 infection. More hospitalized patients had also received a higher cumulative rituximab dose and had an extremely high cumulative dose (≥9000 mg) compared with those without breakthrough infections. Fewer hospitalized patients had received a booster vaccine dose compared with those without any documented COVID-19 infection, except when compared with individuals with mild COVID-19 infections (Table 3).

**Table 3.** Patient Characteristics, SARS-CoV-2 Vaccination, and Rituximab Use Characteristics Among Vaccinated People With Multiple Sclerosis Treated With Rituximab Stratified by COVID-19 Outcomes^a^

Characteristic	No COVID-19 (n = 1232)	Mild COVID-19 (n = 257)	Hospitalized for COVID-19 (n = 27)	Total (N = 1516)	*P* value
Demographic and clinical characteristics^b^					
Age, mean (SD), y	47.7 (12.6)	43.6 (10.7)	48.3 (13.6)	47.0 (12.4)	<.001
Sex					
Female	901 (73.1)	196 (76.3)	19 (70.4)	1116 (73.6)	.54
Male	331 (26.9)	61 (23.7)	8 (29.6)	400 (26.4)	
Race and ethnicity					
Asian or Pacific Islander	38 (3.1)	9 (3.5)	1 (3.7)	48 (3.2)	.005
Black	215 (17.4)	36 (14.0)	3 (11.1)	254 (16.7)	
Hispanic	346 (28.1)	102 (39.7)	13 (48.2)	461 (30.4)	
White	623 (50.6)	109 (42.4)	9 (33.3)	741 (48.9)	
Other^c^	10 (0.8)	1 (0.4)	1 (3.7)	12 (0.8)	
Advanced disability (walker or worse)	209 (17.0)	29 (11.3)	7 (25.9)	245 (16.2)	.03
Elixhauser Comorbidity Index score, median (IQR)	2.0 (1.0-3.0)	2.0 (1.0-3.0)	2.0 (1.0-4.0)	2.0 (1.0-3.0)	.35
Study follow-up time, mean (SD), y	2.1 (0.1)	2.0 (0.2)	1.9 (0.2)	2.1 (0.1)	<.001
COVID-19 vaccination characteristics					
Vaccinated, not boosted	411 (33.4)	154 (59.9)	15 (55.6)	580 (38.3)	<.001
Vaccinated and ≥1 booster or additional dose	821 (66.6)	103 (40.1)	12 (44.4)	936 (61.7)	
No. of additional vaccine doses					
0	411 (33.4)	154 (59.9)	15 (55.6)	580 (38.3)	<.001
1	807 (65.5)	102 (39.7)	12 (44.4)	921 (60.7)	
2	11 (0.9)	1 (0.4)	0	12 (0.8)	
3	3 (0.2)	0	0	3 (0.2)	
Time since last vaccine dose, d					
>150	429 (34.8)	106 (41.2)	7 (25.9)	542 (35.8)	.08
≤150	803 (65.2)	151 (58.8)	20 (74.1)	974 (64.2)	
Time since last vaccine dose, median (IQR), mo	3.7 (2.3-5.8)	4.4 (2.3-7.7)	3.8 (1.3-5.1)	3.8 (2.3-5.9)	.03
Vaccination types					
mRNA vaccines only	1154 (93.7)	242 (94.2)	21 (77.8)	1417 (93.5)	.003
Viral vector vaccines only	55 (4.4)	12 (4.6)	5 (18.5)	72 (4.7)	
Viral vector followed by mRNA booster	23 (1.9)	3 (1.2)	1 (3.7)	27 (1.8)	
Antiviral use					
None	1232 (100)	246 (95.7)	11 (40.7)	1489 (98.2)	<.001
Nirmatrelvir-ritonavir	0	1 (0.4)	0	1 (0.1)	.19
Remdesivir	0	10 (3.9)	16 (59.3)	26 (1.7)	<.001
Rituximab characteristics					
Time since first rituximab dose, median (IQR), y	3.4 (2.3-4.9)	3.3 (2.2-4.5)	3.9 (2.1-6.0)	3.4 (2.3-4.8)	.35
Time since last rituximab infusion, median (IQR), mo	7.0 (3.5-10.9)	5.7 (2.9-8.4)	4.4 (2.0-6.9)	6.7 (3.4-10.3)	<.001
Total No. of rituximab infusions, median (IQR)	5 (3-7)	5 (3-7)	6 (4-11)	5 (3-7)	.21
Dose at last infusion, mg					
<500	15 (1.2)	2 (0.8)	0	17 (1.1)	.63
500	1110 (90.1)	236 (91.8)	23 (85.2)	1369 (90.3)	
>500-<1000	9 (0.7)	0	0	9 (0.6)	
≥1000 mg	98 (8.0)	19 (7.4)	4 (14.8)	121 (8.0)	
Cumulative rituximab dose					
Median (IQR), mg	2500 (2000-4000)	2900 (1800-4000)	3500 (2000-6500)	2500 (2000-4000)	.13
≥9000 mg	69 (5.6)	13 (5.1)	5 (18.5)	87 (5.7)	.01
Timing of SARS-CoV-2 vaccine dose and rituximab					
All doses ≤6 mo from last rituximab dose	203 (16.5)	58 (22.6)	15 (55.6)	276 (18.2)	<.001
Any dose >6 mo from last rituximab dose	1029 (83.5)	199 (77.4)	12 (44.4)	1240 (81.8)	
Time to first vaccine dose from last rituximab dose, median (IQR), mo	8.0 (5.5-13.7)	7.6 (5.2-12.0)	4.9 (3.1-6.4)	7.9 (5.4-13.3)	<.001

Abbreviation: mRNA, messenger RNA.

^a^ Data are presented as number (percentage) of study participants unless otherwise indicated.

^b^ At index date.

^c^ Other race and ethnicity includes American Indian or Alaska Native (n = 3) and multiple races (n = 9).

Receiving at least 1 SARS-CoV-2 vaccine dose more than 6 months after the last rituximab infusion and receiving mRNA vaccines (as opposed to viral vector vaccines) were independently associated with a significantly lower risk of hospitalization for COVID-19. Those with advanced disability were more likely to be hospitalized for COVID-19, although this finding was not statistically significant (Table 2). A higher cumulative rituximab dose was not associated with COVID-19 hospitalization after adjustment for confounders (Table 2).

Sensitivity analyses excluding hospitalizations for patients with COVID-19 who did not require oxygen support (n = 10) decreased the crude incidence race of hospitalization from 2.19 to 1.47 per 100 person-years among vaccinated patients receiving rituximab, although they remained at increased risk of hospitalization compared with the no or other DMT group (adjusted odds ratio [aOR], 6.18; 95% CI, 2.31-16.56).

Individuals receiving rituximab were also more likely to have a mild case of breakthrough COVID-19 infection documented in their EHR compared with the no or other DMT group during the study period (Table 1). Among individuals receiving rituximab, those with documented mild COVID-19 were younger, less likely to be White, had less MS-related disability, and were less likely to have received a booster vaccine dose but had similar rituximab use characteristics compared with those with no documentation of any COVID-19 infection (Table 3). Most mild breakthrough infections among individuals receiving rituximab occurred during the Omicron variant outbreak (n = 209 [81.3%]).

During the study period, 437 individuals receiving rituximab (22.4%) were unvaccinated. These patients were at significantly higher risk of COVID-19 hospitalizations (26 [5.9%], including 2 deaths) compared with vaccinated individuals (aOR, 4.07; 95% CI, 2.29-7.24; *P* < .001) (eTable 2 in Supplement 1). The crude incidence rate of hospitalization for COVID-19 was markedly higher in unvaccinated individuals receiving rituximab (3.56 per 100 person-years) compared with mRNA-vaccinated individuals receiving rituximab (1.81 per 100 person-years), and both of these rates were higher than for mRNA-vaccinated individuals in the no or other DMT group (0.33 per 100 person-years) (Figure). Unvaccinated individuals receiving rituximab were also more likely to experience mild COVID-19 infections (n = 190 [43.5%]) compared with vaccinated individuals (n = 257 [17.0%]).

**Figure.**
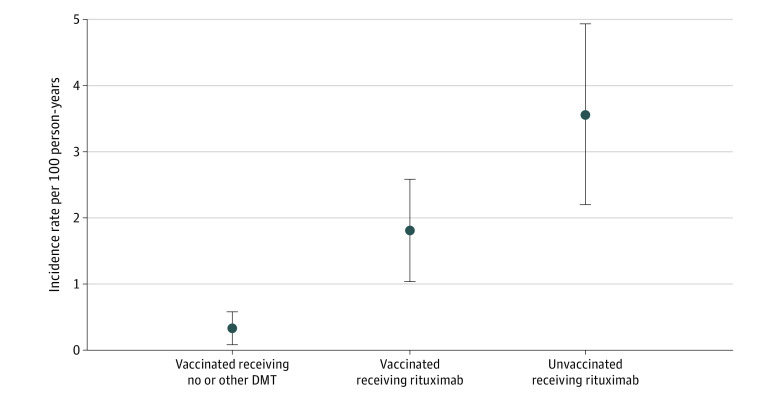
SARS-CoV-2 Messenger RNA (mRNA) Vaccination, Rituximab Treatment, and the Incidence of COVID-19 Hospitalizations Among Persons With Multiple Sclerosis (MS) The figure shows the crude incidence rates of hospitalization for COVID-19 and 95% CIs (error bars) among persons with MS who received 2 doses of mRNA vaccines against SARS-CoV-2 who were untreated or treated with disease-modifying therapies (DMTs) that do not interfere with vaccine efficacy (vaccinated receiving no or other DMT, n = 2458), were treated with rituximab and received 2 doses of mRNA vaccines regardless of time since last rituximab infusion (n = 1516), or were treated with rituximab but did not receive any SARS-CoV-2 vaccine doses (n = 437) following the date of first vaccination (December 15, 2020). Individuals in the vaccinated receiving no or other DMT group and the vaccinated receiving rituximab group received mRNA SARS-CoV-2 vaccines. The crude incidence of hospitalization is low in all groups yet significantly higher in the vaccinated receiving rituximab group compared with the no or other DMT group and highest among the unvaccinated receiving rituximab group.

## Discussion

In this retrospective cohort study, rituximab treatment was associated with reduced SARS-CoV-2 vaccine efficacy among individuals with MS, particularly among those who were vaccinated within 6 months following a rituximab infusion compared with those who received at least 1 vaccine dose more than 6 months after their most recent infusion. Additionally, more individuals receiving rituximab required hospitalization for breakthrough infections and tested positive in the outpatient setting compared with individuals with MS who were untreated or treated with DMTs that do not interfere with vaccines. Paralleling literature in the general population,^15^ we also found that SARS-CoV-2 vaccines were associated with a reduced risk of hospitalization or death from COVID-19 among individuals receiving rituximab, mRNA vaccines were associated with a lower risk of hospitalization than viral vector vaccines, booster doses were associated with a further decreased risk, and mild breakthrough infections were more common during the Omicron than Delta outbreak. The crude incidence rates of COVID-19 hospitalizations were low in mRNA-vaccinated individuals receiving rituximab, but this rate was still higher than in the no or other DMT group, and no deaths were observed in vaccinated individuals with MS. Taken together with the existing literature,^4,5,16^ these findings imply that the increased risk of severe COVID-19 infections associated with B-cell–depleting therapies in persons with MS can be alleviated by (1) getting vaccinated, preferably with an mRNA vaccine; (2) receiving additional booster doses as per public health guidelines; and (3) extending dosing intervals beyond every 6 months to allow for B-cell repopulation of 40/μL or more before administration of vaccines whenever possible.

Our findings are consistent with a carefully conducted prospective cohort study by Sormani et al^5^ that tracked virus-specific serum antibody titers and breakthrough COVID-19 infections following mRNA vaccination in persons with MS, including 272 individuals treated with ocrelizumab and 48 treated with rituximab. That study, conducted during the Delta and initial Omicron outbreaks, reported rates of breakthrough COVID-19 infections among persons receiving B-cell–depleting therapies (19.1%)^5^ that were similar to the rate found in the present study (18.8%), with a very low hospitalization rate (1.1%)^5^ that was similar to the present study’s rate for mRNA-vaccinated individuals receiving rituximab (1.8%); that study also reported no deaths.^5^ As in our study, these rates were significantly higher than for people with MS treated with other DMTs, with the exception of fingolimod, which was associated with an increased risk of breakthrough COVID-19 infections.^5^ Sormani et al^5^ found that breakthrough COVID-19 infections directly corresponded to lower virus-specific antibody titers, due to impaired vaccine response and waning of titers over time. Similar to our findings, Sormani et al^5^ reported that a third mRNA vaccine dose was associated with a reduced risk of breakthrough infections. Neither that study^5^ nor the 2 other studies^6,7^ examining breakthrough COVID-19 infections following vaccination in people with MS were able to examine whether extending rituximab or ocrelizumab dosing intervals beyond 6 months ameliorated this risk.

Our finding that extending rituximab dosing intervals beyond every 6 months to maximize vaccine efficacy resulted in fewer breakthrough COVID-19 infections and hospitalizations compared with vaccinations administered within 6 months of the last infusion is consistent with SARS-CoV-2 vaccine response studies^4,16^ and B-cell depletion dynamics. The humoral response to SARS-CoV-2 vaccines depends on the peripheral B-cell counts, with impaired responses when B-cell counts are below 40 cells/μL and normal responses when counts are 40 cells/μL or higher.^16^ The extent and duration of peripheral B-cell depletion depend on dose and dosing frequency, and counts are typically continuously below 40/μL in the majority of people with MS treated with the US Food and Drug Administration–approved dose of ocrelizumab^17^ (600 mg every 6 months) and commonly used rituximab dosing regimens of 1000 mg or 500 mg every 6 months.^18^ The duration of B-cell depletion is also longer with higher doses and repeated courses of treatment^19^ and shorter when antirituximab antibodies are present^18^—determined with a test that is not commercially available. Although it would be ideal to ensure that CD19^+^ cell counts were 40 cells/μL or greater before vaccination rather than relying on rituximab dosing intervals as a proxy, we were unable to examine this factor due to missing CD19^+^ cell counts, data that were not missing at random. We think it is reasonable to assume that most of our patients vaccinated after 6 months had some B-cell repletion because our cohort was treated predominantly with 500 mg and reduced to annual infusions at the beginning of the pandemic.^1^

The political polarization of COVID-19 vaccines in the US has resulted in approximately 27% of Californians remaining unvaccinated,^20^ paralleling our population of people with MS treated with rituximab in which 22% also remained unvaccinated. Unsurprisingly, these unvaccinated individuals were at a significantly higher risk of contracting and requiring hospitalization for COVID-19, including 2 deaths, compared with vaccinated individuals. Of note, vaccination is the most important factor in preventing hospitalization and death due to COVID-19, including among individuals with MS undergoing rituximab treatment. These outcomes also serve as an important reminder that improving access to SARS-CoV-2 vaccines for those who reside in low- and middle-income countries, where rituximab use is rapidly increasing, needs to be prioritized.

### Strengths and Limitations

This study has several strengths, including the large number of individuals with MS taking rituximab, the population-based source, and the comprehensive EHRs that include comorbidities, COVID-19 infection, and vaccination status. These comprehensive records allowed us to distinguish between which people with MS with positive COVID-19 test results were hospitalized for COVID-19 or unrelated reasons, an increasingly common scenario. Although disconcerting, another strength was our ability to compare outcomes directly with unvaccinated people with MS during the Delta and Omicron outbreaks, demonstrating the importance of vaccination in preventing infection, hospitalization, and death from COVID-19. In addition, the relatively common practice of extending rituximab dosing intervals beyond 6 months in our group allowed us to examine its effects on clinical manifestations of vaccine efficacy. Although some clinicians may fear that extending rituximab or ocrelizumab dosing intervals may result in return of MS disease activity, there is no evidence that continuous B-cell depletion is required to maintain MS disease control,^21^ no reports of return of B-cells rapidly precipitating an MS relapse,^22^ and no reports of rebound disease activity following rituximab or ocrelizumab dose extensions or cessation.^23,24,25,26,27^

This study has some limitations. The small number of hospitalizations and no deaths among vaccinated persons with MS, although reassuring, are the biggest limitations of this study, and the estimated magnitudes of effect should be interpreted with great caution. For this reason, we also cannot exclude the possibility that a small increased risk of death among vaccinated persons with MS taking rituximab remains or that cumulative rituximab dose is an independent risk factor for COVID-19 hospitalizations. The use of self-identified race and ethnicity as a proxy measure for structural racism and the resultant lower socioeconomic status rather than direct measures of experienced structural racism and socioeconomic status is also a weakness. At-home antigen tests became widely available during the Omicron variant outbreak; it is possible that persons with MS taking rituximab may have tested more frequently and were more likely to report a positive at-home test result, and it is possible that physicians were more likely to record the result because of the known increased risk of more severe COVID-19 in unvaccinated persons with MS taking rituximab. Other limitations include the rare use of antivirals and small number of patients with more than 1 booster vaccine dose, precluding our ability to examine the potentially protective effects of these factors. Although we cannot draw conclusions from this study, it seems prudent to recommend complying with public health recommendations for additional vaccine doses in immunocompromised individuals and prescribing nirmatrelvir-ritonavir to patients with COVID-19 and MS who are taking rituximab. Both of these interventions are likely to reduce the risk of morbidity from COVID-19 in patients taking rituximab even further. Finally, whether these results can be extrapolated to the current Omicron variant for which recurrent mild infections in healthy, vaccinated individuals is common, or any future strain, is uncertain.

## Conclusions

In this retrospective cohort study, the absolute risk of hospitalization for COVID-19 among vaccinated persons with MS receiving rituximab was low, although higher than among vaccinated persons with MS who were untreated or treated with DMTs that do not interfere with vaccine efficacy. This risk may be mitigated by using mRNA vaccines, receiving booster vaccine doses, and extending rituximab dosing intervals to allow for vaccination more than 6 months after the last infusion. To ensure full vaccine efficacy, waiting until CD19^+^ cell counts are greater than 40 cells/μL before vaccination appears advisable, although this may not be feasible in many settings.

Rituximab biosimilars have marked affordability, efficacy, and convenience advantages over other DMTs, particularly in countries with poor access to MS specialty care and unaffordable drug prices, including the US.^28^ The low risk of hospitalization for COVID-19 among mRNA-vaccinated individuals with MS receiving rituximab should not preclude rituximab use. Instead, expanding access to SARS-CoV-2 vaccines for individuals receiving rituximab therapy in low- and middle-income countries should be prioritized.

## Notes

Supplement 1. **eMethods.** Supplemental Methods **eTable 1.** SARS-CoV-2 Variants Stratified by COVID-19 Outcomes Among Vaccinated MS Patients **eTable 2.** Association of SARS-CoV-2 Vaccination With COVID-19 Hospitalization Among RTX-Treated Persons With MS

Supplement 2.
Data Sharing Statement
