# Supplementary material for: Analysis of Rituximab Use, Time Between Rituximab and SARS-CoV-2 Vaccination, and COVID-19 Hospitalization or Death in Patients With Multiple Sclerosis
Source: JAMA Netw Open. 2022 Dec 28;5(12):e2248664. doi: 10.1001/jamanetworkopen.2022.48664 (PMC9857265; doi:10.1001/jamanetworkopen.2022.48664)
Supplement: Supplement 1. — eMethods. Supplemental Methods eTable 1. SARS-CoV-2 Variants Stratified by COVID-19 Outcomes Among Vaccinated MS Patients eTable 2. Association of SARS-CoV-2 Vaccination With COVID-19 Hospitalization Among RTX-Treated Persons With MS [file jamanetwopen-e2248664-s001.pdf]

Supplementary Online Content

Smith JB, Gonzales EG, Li BH, Langer-Gould A. Analysis of rituximab use, time between rituximab and SARS-CoV-2 vaccination, and COVID-19 hospitalization or death in patients with multiple sclerosis. *JAMA Netw Open*. 2022;5(12):e2248664. doi:10.1001/jamanetworkopen.2022.48664

eMethods. Supplemental Methods

eTable 1. SARS-CoV-2 Variants Stratified by COVID-19 Outcomes Among Vaccinated MS Patients

eTable 2. Association of SARS-CoV-2 Vaccination With COVID-19 Hospitalization Among RTX-Treated Persons With MS

This supplementary material has been provided by the authors to give readers additional information about their work.

eMethods. Supplemental Methods

Primary Outcome Measure: The primary outcome was defined *a priori* as either requiring hospitalization or death due to COVID-19. As no COVID-19 related deaths were observed, the analyses reflect risk of hospitalization only.

Details of Exclusion Criteria: Treatment with DMTs during the study period known or suspected to interfere with vaccine efficacy (fingolimod n=65, teriflunomide n=3, and azathioprine n=9); treatment with other B-cell depleting agents (ocrelizumab=0, ofatumumab n=3); or partial vaccination defined as only one dose of a mRNA vaccine or SARS-CoV-2 infection occurred within 14 days of the 2nd dose of the initial mRNA vaccine series (n=65).

Definition of Disease Modifying Therapy Use: Rituximab use was defined as at least one infusion within 2 years prior to receiving the first vaccine dose. Use of the other disease modifying therapies was defined as at least 4 infusions of natalizumab or 3 months of dispensed prescriptions for glatiramer acetate, beta-interferons, or dimethyl fumarate within 1 year prior to the first vaccine dose.

Definition of boosted: We defined receiving a booster vaccination as at least 1 additional vaccine dose after completion of the initial vaccination series (2 mRNA vaccines or 1 viral vector vaccine).

Power to detect difference in COVID-19 Mortality Rate: Under the hypothesis that rituximab treatment significantly interferes with vaccine efficacy, then the expected COVID-19 mortality rate should be similar to our unvaccinated rituximab-treated patients, which was 0.24% when alpha was the dominant strain and 0.42% when delta dominated. Based on type 1 error 0.05 and two tailed test, the study is powered on 80.6% for an expected mortality of 0.42% and 57.8% for expected mortality of 0.24%.

© 2022 Smith JB et al. *JAMA Network Open.*

eTable 1. SARS-CoV-2 Variants Stratified by COVID-19 Outcomes Among Vaccinated MS Patients

	No COVID-19	Mild COVID-19	Hospitalized For COVID-19
	**n=3476**	**n=464**	**n=34**
**Outcomes per variant, n (%)**			
Alpha *(12/15/2020-5/31/2021)*			
No/other DMT	2449 (99.9)	2 (0.08)	0 (0)
Rituximab	1514 (99.9)	2 (0.1)	0 (0)
Rituximab any vaccine dose >6mos	1238 (99.8)	2 (0.2)	0 (0)
Rituximab all vaccine doses ≤6mos	276 (100)	0 (0)	0 (0)
Delta *(6/1/2021-11/30/21)*			
No/other DMT	2400 (98.2)	41 (1.7)	4 (0.2)
Rituximab	1454 (96.4)	46 (3.1)	9 (0.6)
Rituximab any vaccine dose >6mos	1205 (97.7)	25 (2.0)	3 (0.2)
Rituximab all vaccine doses ≤6mos	249 (90.2)	21 (7.6)	6 (2.2)
Omicron *(12/1/2021-2/15/2022)*			
No/other DMT	2193 (92.9)	164 (7.0)	3 (0.1)
Rituximab	1204 (84.1)	209 (14.6)	18 (1.3)
Rituximab any vaccine dose >6mos	1009 (84.8)	172 (14.5)	9 (0.8)
Rituximab all vaccine doses ≤6mos	195 (80.9)	37 (15.4)	9 (3.7)

Abbreviations: SARS-CoV-2=severe acute respiratory syndrome coronavirus 2; COVID-19=coronavirus disease 2019; MS=multiple sclerosis; DMT=disease-modifying therapies; mos=months

© 2022 Smith JB et al. *JAMA Network Open.*

eTable 2. Association of SARS-CoV-2 Vaccination With COVID-19 Hospitalization Among RTX-Treated Persons With MS*

**Variable**	**Crude OR**	**95% CI**		**p-value**	**Adjusted OR**	**95% CI**		**p-value**
**Age,** years	1.02	0.99	1.04	0.16	1.02	0.99	1.05	0.14
**Female Sex** (ref=male)	0.96	0.53	1.76	0.90				
**Race/ethnicity** (ref=other)								
White	0.71	0.41	1.24	0.23				
**MS Disability** (ref=cane or lower)								
Walker or worse	1.94	1.02	3.67	0.04	1.74	0.85	3.59	0.13
**Elixhauser Comorbidity Index**	1.10	0.95	1.28	0.20	1.08	0.91	1.28	0.41
**Vaccination Characteristics**								
Vaccination status (ref=vaccinated)								
Unvaccinated	3.49	2.01	6.04	<0.001	4.07	2.29	7.24	<0.001
**Rituximab Characteristics**								
Time since last RTX dose (months)	0.95	0.90	1.00	0.03	0.94	0.89	0.99	0.02
Dose at last infusion (ref<1000mg)								
>=1000mg	1.93	0.93	4.02	0.08	1.11	0.49	2.50	0.81
Total cumulative dose (ref<9000mg)								
>=9000mg	3.64	1.73	7.67	<0.001	2.51	1.07	5.87	0.03

*Following availability of vaccines for RTX-MS (12/15/2020) through end of study period (2/15/2022)
Abbreviations: SARS-CoV-2=severe acute respiratory syndrome coronavirus 2; COVID-19=coronavirus disease 2019; RTX=rituximab; MS=multiple sclerosis; OR=odds ratio; 95% CI=95% confidence interval; ref=reference; mg=milligrams

© 2022 Smith JB et al. *JAMA Network Open.*
