# Supplementary material for: Analysis of Rituximab Use, Time Between Rituximab and SARS-CoV-2 Vaccination, and COVID-19 Hospitalization or Death in Patients With Multiple Sclerosis
Source: JAMA Netw Open. 2022 Dec 28;5(12):e2248664. doi: 10.1001/jamanetworkopen.2022.48664 (PMC9857265; doi:10.1001/jamanetworkopen.2022.48664)
Supplement: Supplement 2. — Data Sharing Statement [file jamanetwopen-e2248664-s002.pdf]

Data Sharing Statement

Smith. Analysis of Rituximab Use, Time Between Rituximab and SARS-CoV-2 Vaccination, and COVID-19 Hospitalization or Death in Patients With Multiple Sclerosis. *JAMA Netw Open*. Published December 28, 2022. doi:10.1001/jamanetworkopen.2022.48664

Data
Data available: No

Additional Information
Explanation for why data not available: Due to KPSC's institutional review board, data are available upon reasonable request.
